# Robustness of radiomic features in magnetic resonance imaging: review and a phantom study

**DOI:** 10.1186/s42492-019-0025-6

**Published:** 2019-11-20

**Authors:** Renee Cattell, Shenglan Chen, Chuan Huang

**Affiliations:** 1 Department of Biomedical Engineering, Stony Brook University, Stony Brook, NY 11794 USA; 2 Department of Radiology, Stony Brook Medicine, Stony Brook, NY 11794 USA; 3 Department of Psychiatry, Stony Brook Medicine, Stony Brook, NY 11794 USA

**Keywords:** Radiomics, Robustness, Magnetic resonance imaging, Imaging biomarker, Phantom study

## Abstract

Radiomic analysis has exponentially increased the amount of quantitative data that can be extracted from a single image. These imaging biomarkers can aid in the generation of prediction models aimed to further personalized medicine. However, the generalizability of the model is dependent on the robustness of these features. The purpose of this study is to review the current literature regarding robustness of radiomic features on magnetic resonance imaging. Additionally, a phantom study is performed to systematically evaluate the behavior of radiomic features under various conditions (signal to noise ratio, region of interest delineation, voxel size change and normalization methods) using intraclass correlation coefficients. The features extracted in this phantom study include first order, shape, gray level cooccurrence matrix and gray level run length matrix. Many features are found to be non-robust to changing parameters. Feature robustness assessment prior to feature selection, especially in the case of combining multi-institutional data, may be warranted. Further investigation is needed in this area of research.

## Introduction

### Overview of radiomics

Radiomics is the extraction of high-dimensional and quantitative mineable data from digital medical images [1–3]. The prefix “radio-” refers to the use of radiological images; these digital medical images can come from various modalities, but are most frequently computed tomography (CT), positron emission tomography (PET) and magnetic resonance imaging (MRI) [1, 2]. Patients often receive numerous imaging studies to diagnose, stage, plan treatment and monitor disease progression. Currently in clinical practice, imaging data is only qualitatively or semi-quantitively utilized and a dictated report is created by the radiologist. Radiomic analysis aims to maximize the amount of quantitative information that can be extracted from the existing medical images that may not be appreciable to the naked eye, adding more valuable information that can be used for patient care. The digital image is analyzed by mathematical algorithms and/or filtering of the data to result in a quantitative value. These features are termed quantitative imaging biomarkers. These features can be classified into 2 different groups: semantic and agnostic.

Semantic features can be either qualitatively defined by a radiologist or quantitatively defined by a mathematical algorithm. Examples of semantic features include size, shape, location, vascularity, and spiculation [1, 2]. These are descriptors that are commonly used by radiologists in a qualitative fashion to identify and characterize disease, such as in the case of breast tumors where the size of tumor is indicative of treatment response (Response evaluation criteria in solid tumors criteria) and spiculation being a higher chance of malignancy (Breast Imaging Reporting and Data System) [1, 4–6]. Quantitative extraction of semantic features is desired to give a more comprehensive and reproducible description of the region of interest (ROI), whereas visual inspection by radiologist has large intra- and inter-reader variability [5].

Agnostic features aim to quantify the heterogeneity within a ROI based on image intensity. Agnostic features can be further broken down into first order features, second-order features and higher-order features:

First order features are commonly histogram-based and examine gray level signal intensity within a ROI independent of spatial relationships between adjacent voxels. Examples of these features include uniformity, entropy, mean, median and kurtosis [1, 2].

Second-order features, commonly referred to as “texture” features, examine spatial relationship between gray level signal intensities by constructing a gray-level dependence matrix [1, 2]. These features give a measure of intra-region heterogeneity. These were first explored by Haralick et al. [7] in the advent of gray level cooccurrence matrix (GLCM) by analyzing the occurrence of different gray level voxel pairs in different directions. Over the development of radiomics, these features have expanded to include different ways of quantifying spatial relationship between voxels, such as gray level run length matrix (GLRLM), which quantifies the number of consecutive voxels with same gray level [8], and gray level zone length matrix, which quantifies the size of a homogenous area of an image [9].

Higher-order features involve application of a filter or transformation to an image prior to feature extraction. These features aim to identify patterns or highlight details within the image that are not initially perceivable by the reader or are hard to interpret [1, 5]. An example of this type of feature is wavelet transform [10].

As such, this analysis has exponentially increased the amount of information that can be extracted from a single digital image. A single image may contain valuable sub-visual information of the tissue pathophysiology, phenotype and microenvironment that can be captured by quantitative analysis [2].

The suffix “-omics” refers to the combination of this massive amount of quantitative features that can be extracted from a single ROI using mathematical/statistical methods with clinical characteristics to be used in clinical management of patients [1, 2]. A goal of radiomics is to identify robust and consistent imaging biomarkers to aid in clinical decision making, such as the diagnosis of a disease, monitoring of treatment response or prediction of prognosis [1]. This is a step towards “precision” or “personalized” medicine in which these large number of quantitative features from the image of a specific individual coupled with their individual clinical characteristics (age, genomic profiling, etc.) can be used to tailor treatment or assess risk [1, 2, 5].

A large area of study in the field of radiomics include oncological applications, attributed to Quantitative Imaging Network, funded by National Institutes of Health and the Quantitative Imaging Biomarker Alliance, organized by the Radiological Society of North America [2, 5]. Cancer has been noted to be a highly heterogenous disease on both an inter-patient and intra-patient level [2, 11, 12]. There are many applications of radiomics in oncological applications. There is a need for a non-invasive imaging biomarker to better characterize lesions, such as tumor aggressiveness, because a single needle biopsy cannot capture the entire landscape of a tumor [5]. In the case of a more aggressive tumor, it is possible that a more intensive treatment regimen may be tailored to those patients resulting in an improved prognosis [11]. Additionally, characterizing a lesion as malignant or benign could be a useful tool for clinicians to make a more informed diagnosis, reducing stress for the patient and identifying the correct course of action. Furthermore, radiomic analysis could aid in the monitoring of treatment response; current criteria include mainly size and shape changes, whereas there may be subtle changes in the image appearance, not clinically appreciable to the naked eye, which is informative of response [5, 11]. It is possible, that in the case of a clearly non-responding tumor, the patient may be switched to a different/more effective therapy and avoid side effects associated with a treatment from which they are not expected to receive clinical benefit.

As previously mentioned, radiologic images including CT, PET and MRI have been used in radiomics studies. In this article, we focus on MRI. Each modality has its own characteristics which could affect the radiomic analysis. CT and PET have pixel/voxel values with a physical meaning, namely characterizing the x-ray attenuation of tissue through Hounsfield units and cellular activity through Standard Uptake Value, respectively. Thus, the diagnostic or prognostic implications resulting from radiomic analysis will have variable interpretations.

### Radiomics in MRI

#### Overview

MRI is a commonly used modality for radiomic analysis owing to its’ rich contrast mechanisms (such as T1, T2, chemical exchange, diffusion, perfusion, contrast enhancement) and fine soft-tissue detail [13]. A majority of MRI radiomic analysis is performed in oncological applications such as head and neck, prostate, brain and breast cancer.

##### Head and neck cancer

Numerous studies have performed MRI radiomic analysis on head and neck cancer. Analyzed endpoints included pathological classification, segmentation and prognostic/predictive biomarkers of progression, survival or treatment, with reports of radiomic model performance showing promising results in most studies [13].

##### Prostate cancer

Multiparametric MRI is an important tool in the diagnosis of prostate cancer, with T2-weighted, dynamic contrast enhanced and diffusion weighted imaging being the core imaging sequences in the Prostate Imaging Reporting and Data System [14]. Detection of prostate cancer is the main focus of radiomics as it applies to prostate cancer, specifically with identification and delineation of the tumor region being the priority [15].

##### Brain cancer

MRI is a standard of care for brain tumors, most commonly in the form of the contrast-enhanced imaging which can identify tumor areas through their leaky vasculature and breakdown of the blood brain barrier. Main clinical applications of radiomics in brain cancer include prediction of prognosis (survival time), classification of glioblastoma subtypes and discrimination of radiation necrosis tissue from recurrent tumor tissue [16].

##### Breast cancer

MRI is the modality of choice for assessing extent of disease and monitoring treatment response in patients diagnosed with breast cancer. Similar to brain cancer, a dynamic contrast enhanced series is commonly performed to identify areas of increased, disorganized vascularity associated with malignancy. Studies performed have looked at differentiating benign from malignant lesions, prediction of treatment response, prediction of lymph node metastasis, prediction of molecular profile and prediction of risk of recurrence [17–19].

##### Others

Aside from oncological applications, radiomic analysis has been explored in other pathologies such as Alzheimer’s disease, multiple sclerosis, ischemic stroke and epilepsy [20–23].

#### Steps of MRI radiomics

Radiomic analysis of MRI generally consists of 4 main steps: image acquisition, ROI segmentation, feature extraction and feature selection.

Image acquisition factors include scanner (make, model, field), coil, sequence [sequence type, echo time (TE), repetition time (TR), acceleration, voxel size, bandwidth, etc.] and reconstruction algorithm (parallel imaging, compressed sensing, regularization parameters, coil combination, etc.).

ROI segmentation includes automatic, semi-automatic or manual delineation of the ROI in the image.

Feature extraction includes pre-processing steps (normalization, binning to a defined number of gray levels) and application of mathematical algorithms or filters to calculate the feature within the ROI.

Feature selection and model construction includes reduction techniques to reduce the number of redundant features and selection by means of machine learning (least absolute shrinkage and selection operator, support vector machine, etc.).

Changing parameters at any steps in the process could result in different feature values, and thus lessen the consistent and reliable predictive performance. Although many of the parameters in this pipeline are easy to standardize, some of them suffer from more variabilities in MRI radiomics.

### Feature robustness in MRI radiomics

#### Importance of robustness of features in medical imaging

A fundamental requirement to draw reliable conclusions based on any radiomics imaging biomarker is that its value must be stable under different conditions and two measurements obtained under the same conditions must be consistent [24]. There is currently no consensus on how to assess the robustness [25–30] (others may refer to it as “stability” [31–36],“reproducibility” [26, 37–40] or “repeatability” [24, 38, 41]) of radiomic features. However, it is recommended in image biomarker standardization initiative (IBSI) [42] to perform feature robustness assessment prior to feature selection. It should be noted that robustness is not a guarantee of the features’ discriminative power and the predictive performance should be investigated [24]. Moreover, feature robustness could be application dependent [43], meaning that a feature that is found to be highly precise for a certain dataset/disease could have poor stability when assessed for another dataset/disease. Several studies [24, 28, 32, 37] emphasized that feature pre-selection based on stability should be performed to generate more reliable results and reduce data dimensionality.

#### Robustness analysis in MRI

Most of the existing publications assessing image biomarker robustness investigated radiomic features from CT and PET images [30, 44–48]. It was stated in a review paper in 2016 [49] that “*the repeatability of MR-based radiomic features has not been investigated*”. Since then, there have been some studies in recent publications investigating the robustness of MRI radiomic analysis, but, due to lack of standardization, frequently leads to inconsistent conclusions. We performed a literature search on peer-reviewed full-text articles that analyzed feature robustness based on MRI and summarized them in Table 1 (16 on human subjects, and 5 exclusively on phantoms). These publications have assessed some parameters such as vendor [33, 40, 51], scanner [31, 33], acquisition parameters [52, 59], observers [26, 37, 39, 50] and pre-processing parameters [24, 38, 50, 53, 54], however, there still remains much to be investigated.

**Table 1:** Summary of literature for magnetic resonance imaging radiomics feature robustness

Reference	Disease / phantom	MR sequences	# features	Feature classes	Parameters evaluated	Statistical analysis	Robustness evaluation
Baessler et al. [26], 2019	Vegetable/fruit phantom	FLAIR, T1w, T2w	45	Intensity, shape, texture	MR sequence, resolution	CCC, DR, Bland-Altman analyses, ICC	Test-retest robustness, intraobserver and interobserver reproducibility
Traverso et al. [50], 2019	Locally advanced rectal cancer	DWI (ADC map)	70	Intensity, shape, texture	Pre-processing filter, re-binning and resampling	CCC, ICC, Spearman correlation	Inter-observer dependence
Duron et al. [39], 2019	Lacrymal gland tumor and breast lesion	T1w, DWI (ADC map), DIXON, DISCO	69/57 (2 softwares)	Texture	Discretization method, bin width and bin number	CCC, ICC(2,1)	Intra- and inter-observer reproducibility
Lecler et al. [37], 2019	Lacrimal gland tumor	T1w, DWI (ADC map), DIXON	85	Intensity, shape, texture	MR sequence, metric threshold	CCC, ICC(2,1), Spearman correlation	Intra- and inter-observer reproducibility, non-redundancy
Um et al. [51], 2019	Glioblastoma multiforme	FLAIR, T1w, post-contrast T1w	420	Intensity, shape, texture, filter-based	Preprocessing technique on multi-scanner datasets, bin number	Two-sided Wilcoxon tests	Feature variability
Schwier et al. [24], 2019	Prostate cancer	T2w, DWI (ADC map)	NA	Intensity, shape, texture, filter-based	Image normalization, 2D/3D texture computation, bin widths, and image pre-filtering	ICC(1,1)	Test-retest repeatability
Fiset et al. [38], 2019	Cervical cancer	T2w	1761	Intensity, shape, texture, filter-based	Quantization method, LoG kernel sizes,	ICC(1,1), ICC(2,1), Pearson correlation, Krippendorff’s alpha	Test-retest repeatability, cross-scanner reproducibility, inter-observer reproducibility
Peerlings et al. [33], 2019	Ovarian, lung and colorectal liver metastasis cancer	DWI (ADC map)	1322	Intensity, shape, texture, filter-based	Center and vendor	CCC	Feature stability
Buch et al. [52], 2018	Nonanatomic Gd-DTPA phantom	T1w	41	Intensity, texture, filter-based (Laws)	Magnet strength, flip-angle, number of excitations, scanner platform	Q values	Feature variability
Yang et al. [53], 2018	Simulated data from digital phantom and glioma	T1w, T2w	26	Texture	Noise level, acceleration factor, and image reconstruction algorithm	Student’s t-test, CV	Feature variance
Bologna et al. [32], 2018	Soft tissue sarcoma and oropharyngeal cancer	DWI (ADC map)	69	Intensity, texture	ROI transformation and bin number	Absolute percentage variation, two-way mixed effect ICC	Feature stability and discrimination
Chirra et al. [40], 2018	Prostate cancer	T2w	406	Intensity, texture, filter-based	Different sites	Multivariate CV and Instability Score	Cross-site reproducibility
Saha et al. [31], 2018	Breast cancer	DCE-MRI (first postcontrast, PE, SER, washing rate maps)	529	Intensity, shape, texture	Scanner, contrast agent	ICC(3,1), Pearson correlation, average DSC	Inter-reader stability, inter-relations within feature groups, pairwise reader variability
Molina et al. [27], 2017	Glioblastoma	T1w	16	Texture	Spatial resolution and bin number	CV	Feature variation
Brynolfsson et al. [54], 2017	Glioma and prostate cancer	DWI (ADC map)	19	Texture	noise level, resolution, ADC map construction, quantization method, and bin number	Two-sample Kolmogorov-Smirnov tests	Feature distribution variation
Gourtsoyianni et al. [41], 2017	Primary rectal cancer	T2w	46	Intensity, texture, filter-based	2 baseline examinations	wCV	Test-retest repeatability
Guan et al. [55], 2016	Cervical cancer	DWI (ADC map)	8	Intensity, texture	GLCM direction	ICC, Wilcoxon test, Kruskal-Wallis test, and ROC curve	Inter- and intra-observer agreement
Molina et al. [56], 2016	Glioblastoma	T1w	16	Texture	Matrix size and bin number	CV	Feature variation
Savio et al. [57], 2010	Multiple sclerosis	T1w	264	Intensity, texture, filter-based	Global, regional and local features	Wilcoxon’s signed ranks test	Feature variation
Mayerhoefer et al. [58], 2009	PSAG phantom	T2w	NA	Texture, filter-based	Spatial resolution, NAs, TR, TE, and SBW	LDA and k-NN classifier	Ability to distinguish between different patterns
Collewet et al. [59], 2004	Cheese phantom	T2w, PDW	90	Texture, filter-based	MRI acquisition protocol and quantization method	POE, ACC, 1-NN classifier	Classification

*MR* Magnetic resonance, *FLAIR* Fluid-attenuated inversion recovery, *DWI* Diffusion-weighted imaging, *ADC* Apparent diffusion coefficient, *DISCO* Differential subsampling with cartesian ordering, *DCE-MRI* Dynamic contrast-enhanced magnetic resonance imaging, *PE* Peak enhancement, *SER* Signal enhancement ratio, *PDW* Proton density weighted, *LoG* Laplacian of Gaussian, *NAs* Number of acquisitions, *TR* Repetition time, *TE* Echo time, *SBW* Sampling bandwidth, *CCC* Concordance correlation coefficient, *DR* Dynamic range, *ICC* Intraclass correlation coefficient, *wCV* Within-subject coefficient of variation, *ROC* Receiver operating characteristic, *CV* Coefficient of variation, *DSC* Dice similarity coefficients, *LDA* Linear discriminant analysis, *k-NN* k nearest neighbor, *POE* Probability of error, *ACC* Average correlation coefficient, *1-NN* 1-nearest neighbor

The importance of complete and clear reporting was also highlighted in several studies. IBSI [42] presented informative reporting guidelines on image pre-processing and feature extraction. Additionally, the radiomics quality score was proposed by the D-Lab [43]; this assigns a value based on 16 key points on the reporting of radiomics studies. With the aid of these two standards, it was found that many studies were lacking in the clear and concise description of (1) software implementation (i.e., chosen setting parameters, equations), (2) pre-processing steps (i.e., normalization, quantization) and (3) statistical methods use to quantify or assess feature robustness [i.e., form of intraclass correlation coefficient (ICC)]. Additionally, use of an external validation set is an important step to robustness feature analysis that was lacking in many of these studies.

**Fig. 1:**
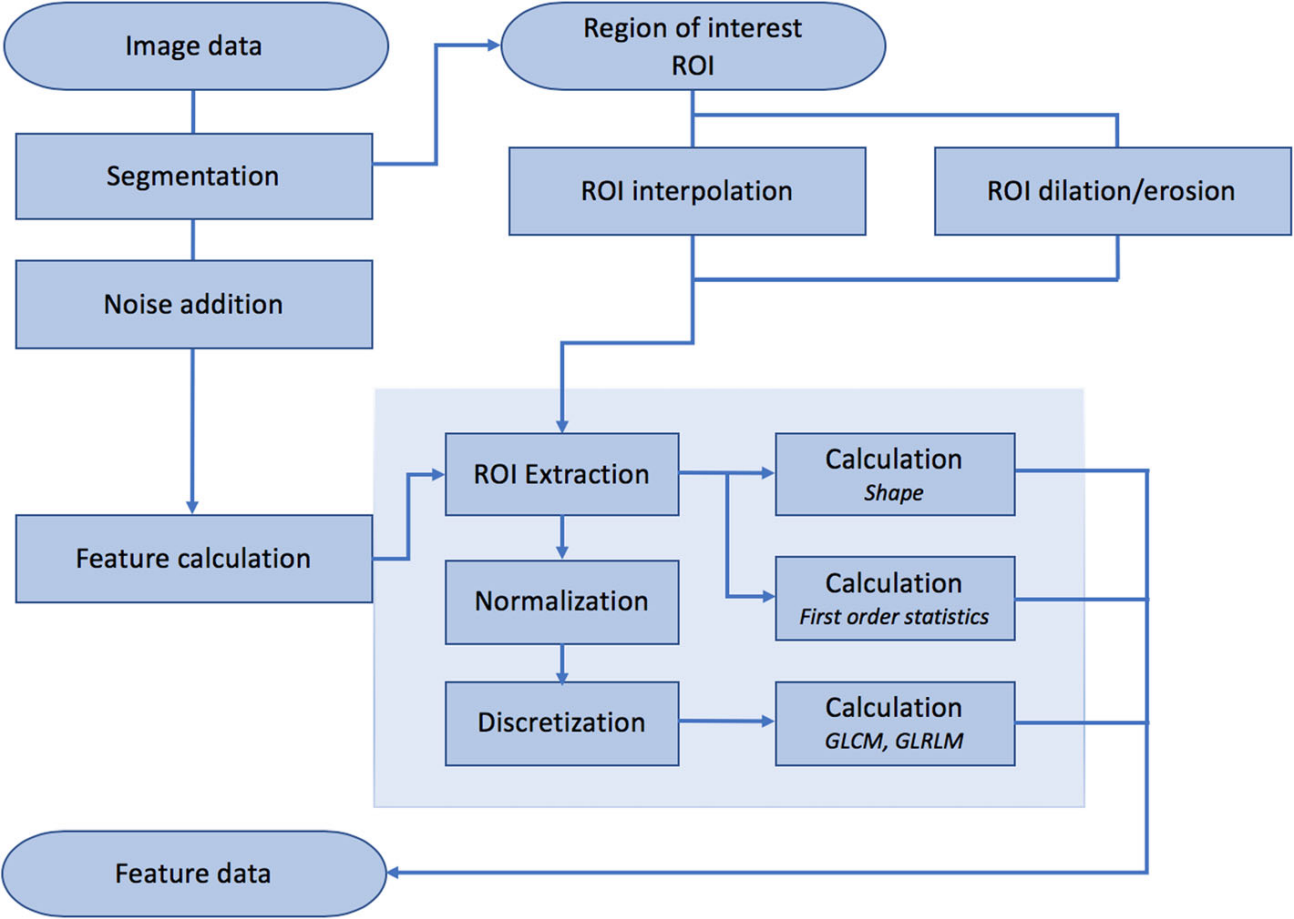
Schematic representation of workflow in this study. Image segmentation is performed manually on a single image. The ROIs are interpolated to images of different in-plane resolutions for voxel size analysis. Gaussian noise is added to generate different signal to noise ratio steps and generate 10 different noise realizations for test-retest analysis. Shape, first order, GLCM and GLRLM features are calculated for each ROI. GLCLM and GLRLM features are calculated after normalization (mean ± 3SD or zero to maximum) and discretization (64 gray levels). *ROI* Region of interest, *GLCM* Gray level cooccurrence matrix, *GLRLM* Gray level run length matrix

We believe one option to improve robustness analysis of MRI radiomics studies is to systematically evaluate the behavior of the radiomic features under various conditions. With a well-defined “dictionary” of robust features, researchers can perform a pre-selection step based on their specific application. Here, we demonstrate such effort by evaluating feature robustness to MRI image signal to noise ratio (SNR), ROI delineation, small voxel size variation and normalization methods through a phantom study. The workflow of the study is displayed in Fig. 1. We measure degree of robustness using ICC (2-way mixed-effects model, single rater, absolute agreement) and separation into three groups based on ICC values: high (> 0.9), moderate (0.5–0.9) and low (< 0.5) for each of the conditions investigated.

## Results and discussion

### SNR

In MRI, there are many factors affecting the SNR of an image even if all acquisition parameters are set to the same values and acquisitions are performed on the same scanner. Examples of these factors include coil load, analog-to-digital gain, shimming, reconstruction method and size of the patient. In fact, due to the inhomogeneity of coil sensitivity, SNR can even vary within the same slice of image. This can be due to both B1+ (transmit) and B1- (receiving) properties of the coil. In this study, we systematically evaluate the effect of several levels of SNR using phantom data with added Gaussian noise. We also analyze the effect of two normalization methods on the radiomic results.

**Fig. 2:**
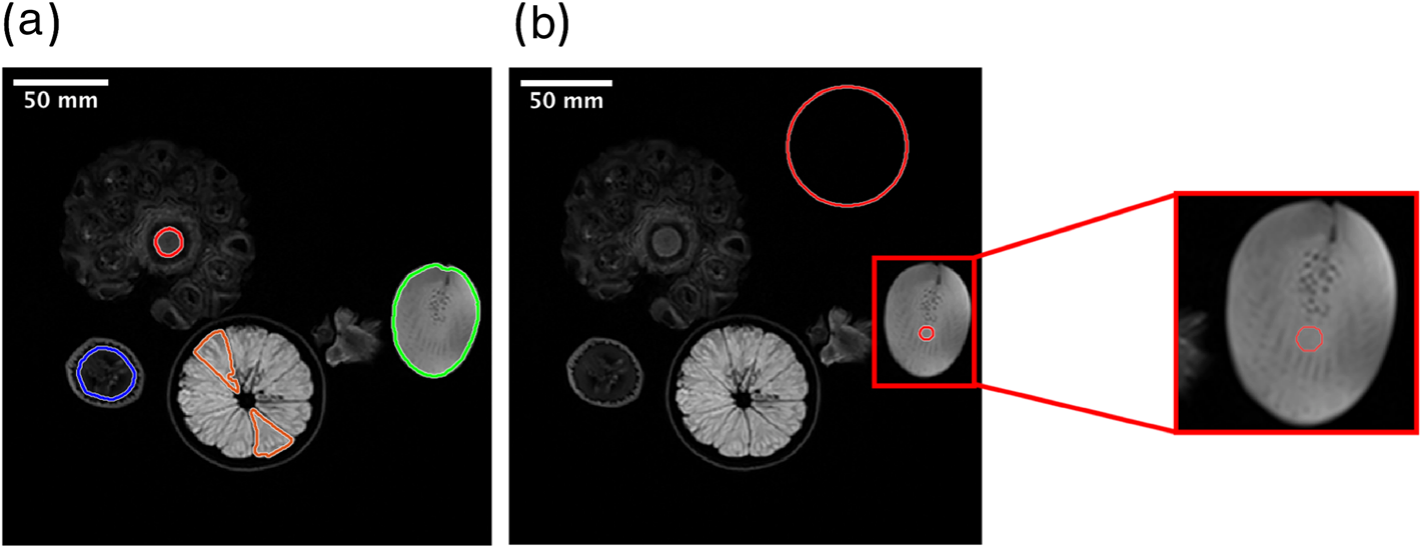
Image of (**a**) regions of interest under investigation in this study, namely pineapple core (red), banana (blue), orange (orange) and kiwi (green), and (**b**) regions of interest used for signal to noise ratio calculation

T2 weighted phantom images used in the analysis are shown in Fig. 2a, with ROIs drawn on a pineapple core (red), banana (blue), orange (orange) and kiwi (green). Regions of interest used in SNR calculation are shown in Fig. 2b.

**Fig. 3:**
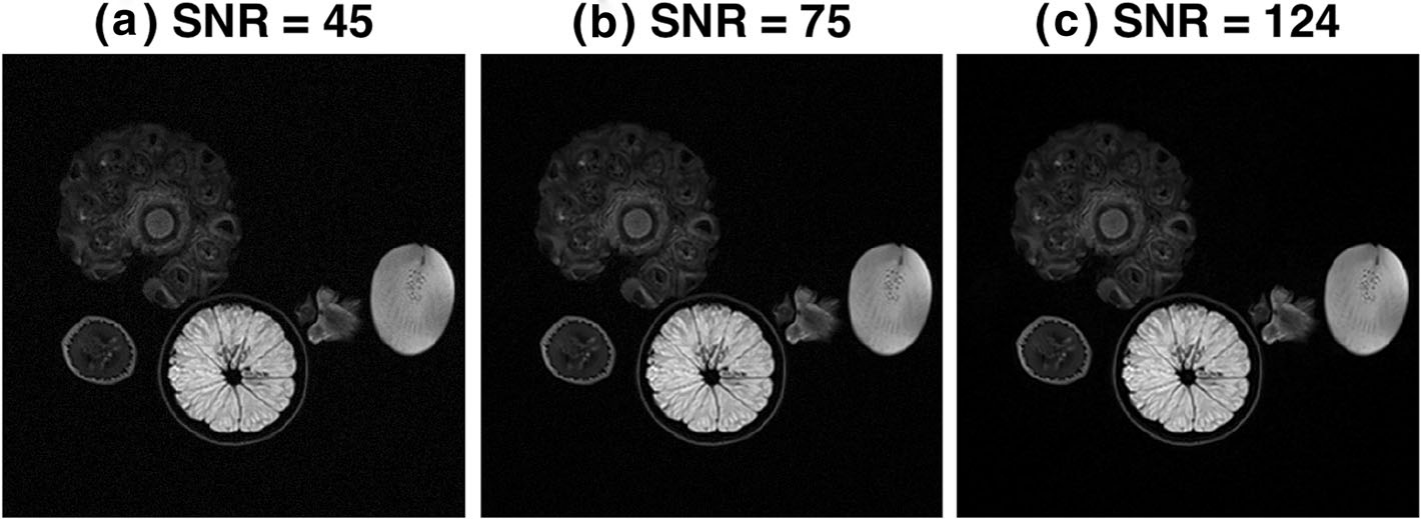
Magnitude images at different signal to noise ratio (SNR) steps: (**a**) SNR = 45, (**b**) SNR = 75 and (**c**) SNR = 124

Complex Gaussian noise was added to the original image (Fig. 3c) and magnitude images were used for the analysis. Two noise levels [SNR 45 (Fig. 3a) and SNR 75 (Fig. 3b)] were generated from the original image whose SNR is 124. To the naked eye, there isn’t a large visual difference between SNR of 45 and SNR of 75. These SNR levels are representative of those seen in clinical images. As mentioned above, SNR is spatially varying in MRI, the SNR values used here are simply representation of the overall noise level of the image.

**Fig. 4:**
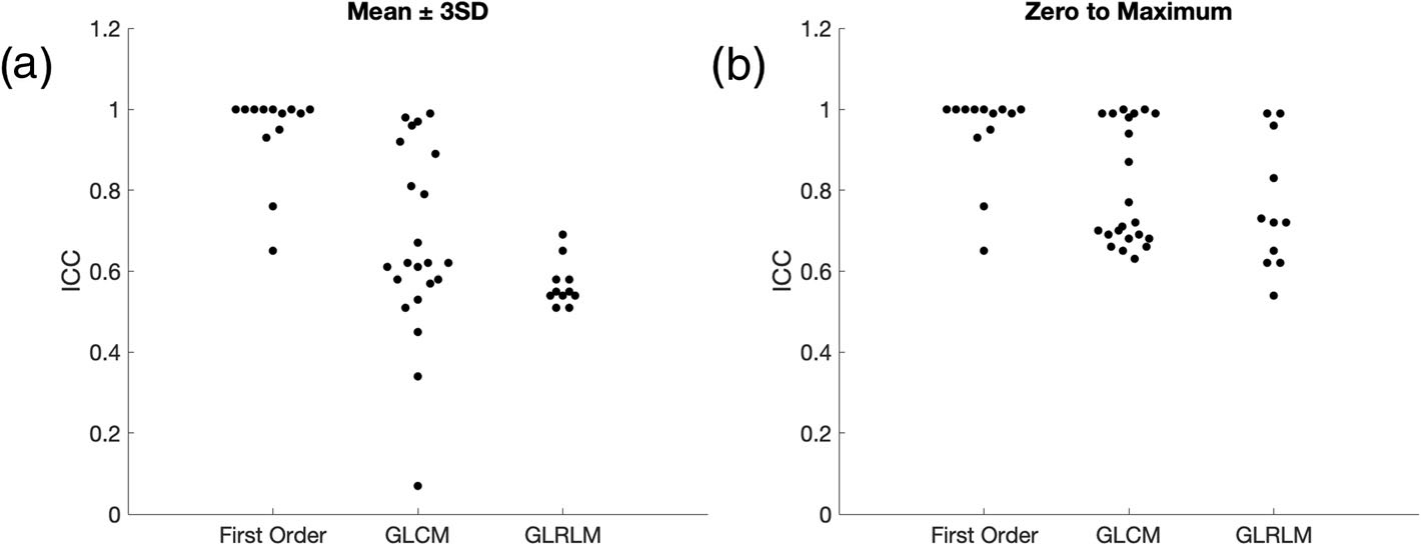
Average intraclass correlation coefficient over 10 noise realizations of first order, GLCM and GLRLM features by using (**a**) mean ± 3SD and (**b**) zero to maximum normalization for signal to noise analysis.* ICC* Intraclass correlation coefficient, *GLCM* Gray level cooccurrence matrix, * GLRLM* Gray level run length matrix

Shape features were omitted from this part of the analysis because the same ROI was used across all SNR steps. This portion of the study aimed to analyze only the effect of added noise, and not intra- or inter-reader variability in ROI delineation. Details of the study is described in the Methods section, summarily, three most commonly used types of features (first order features, GLCM features, and GLRLM features) were studied using 10 different noise realizations and 2 different normalization techniques. Specifically, features within each group and their respective ICCs (2-way mixed-effects model, single rater, absolute agreement) are summarized in Table 2. The results using the first normalization technique (mean ± 3SD) are shown in Table 3 and Fig. 4a. The majority of first order features, 11 out of 13 have an ICC greater than 0.9, indicating high robustness to added noise. However, only 5 out of 22 GLCM features have an ICC greater than 0.9. A majority of the GLCM features (14 out of 22) were found to be of moderate robustness, represented by ICC between 0.5 and 0.9. All GLRLM features were found to have moderate robustness (0.5–0.9).

**Table 2:** Average of intraclass correlation coefficient value over 10 noise realizations in reference to variation in signal to noise ratio, region of interest dilation/erosion and small variation in voxel size

	Normalization	Mean ± 3SD				Zero to maximum			
		SNR	ROI erosion	ROI dilation	Voxel size	SNR	ROI erosion	ROI dilation	Voxel size
First order (*n* = 13)	Energy	**1.00**	**0.99**	**1.00**	0.87	**1.00**	**0.99**	**1.00**	0.87
	Kurtosis	**0.95**	**0.97**	0.78	0.88	**0.95**	**0.97**	0.78	0.88
	Maximum	**1.00**	**0.99**	**1.00**	**0.99**	**1.00**	**0.99**	**1.00**	**0.99**
	Mean deviation	**0.99**	**0.99**	**0.98**	**0.99**	**0.99**	**0.99**	**0.98**	**0.99**
	Mean	**1.00**	**1.00**	**1.00**	**1.00**	**1.00**	**1.00**	**1.00**	**1.00**
	Median	**1.00**	**1.00**	**1.00**	**1.00**	**1.00**	**1.00**	**1.00**	**1.00**
	Minimum	**1.00**	**1.00**	0.78	**1.00**	**1.00**	**1.00**	0.78	**1.00**
	Range	**0.99**	**0.99**	0.81	**0.97**	**0.99**	**0.99**	0.81	**0.97**
	Root mean square	**1.00**	**1.00**	**1.00**	**1.00**	**1.00**	**1.00**	**1.00**	**1.00**
	Skewness	**0.93**	0.81	0.63	0.76	**0.93**	0.81	0.63	0.76
	Variance	**1.00**	**0.99**	**0.95**	**0.98**	**1.00**	**0.99**	**0.95**	**0.98**
	Entropy	0.65	0.77	0.47	0.43	0.65	0.77	0.47	0.43
	Uniformity	0.76	0.86	0.87	0.84	0.76	0.86	0.87	0.84
Shape (*n* = 10)	Mesh surface	N/A	**1.00**	**1.00**	**1.00**	N/A	**1.00**	**1.00**	**1.00**
	Pixel surface	N/A	**1.00**	**1.00**	**1.00**	N/A	**1.00**	**1.00**	**1.00**
	Perimeter	N/A	**0.99**	**1.00**	**1.00**	N/A	**0.99**	**1.00**	**1.00**
	Perimeter to surface ratio	N/A	**0.96**	**0.97**	**0.99**	N/A	**0.96**	**0.97**	**0.99**
	Sphericity	N/A	**0.99**	**0.99**	**0.99**	N/A	**0.99**	**0.99**	**0.99**
	Spherical disproportion	N/A	**0.99**	**0.99**	**0.99**	N/A	**0.99**	**0.99**	**0.99**
	Maximum 2D diameter	N/A	**1.00**	**1.00**	**1.00**	N/A	**1.00**	**1.00**	**1.00**
	Major axis length	N/A	**0.99**	**0.99**	**1.00**	N/A	**0.99**	**0.99**	**1.00**
	Minor axis length	N/A	**0.99**	**0.99**	**1.00**	N/A	**0.99**	**0.99**	**1.00**
	Elongation	N/A	**1.00**	**1.00**	**1.00**	N/A	**1.00**	**1.00**	**1.00**
GLCM (*n* = 22)	Autocorrelation	**0.97**	**0.99**	0.43	**0.90**	**0.99**	**0.98**	**0.99**	**0.98**
	Cluster prominence	0.79	**0.92**	**0.95**	0.89	**1.00**	**1.00**	**1.00**	**0.99**
	Cluster shade	**0.96**	0.68	**0.95**	0.84	**0.94**	0.69	0.90	0.75
	Cluster tendency	0.81	**0.98**	0.79	0.89	**1.00**	**0.99**	**0.99**	**0.99**
	Contrast	0.61	**0.93**	**0.93**	0.90	0.68	**1.00**	**0.95**	**0.94**
	Correlation	0.62	**0.94**	**0.93**	0.90	0.63	**0.93**	**0.93**	0.90
	Difference entropy	0.67	**0.98**	**0.98**	**0.96**	0.77	**1.00**	**0.95**	**0.98**
	Dissimilarity	0.62	**0.95**	**0.98**	**0.93**	0.70	**1.00**	**0.96**	**0.96**
	Energy	0.34	**0.96**	**0.99**	**0.92**	0.72	**0.99**	**0.97**	**0.99**
	Joint entropy	0.45	**0.97**	**0.96**	**0.91**	0.87	**0.99**	**0.97**	**0.99**
	Inverse difference	0.58	**0.98**	**1.00**	**0.94**	0.69	**1.00**	**0.98**	**0.97**
	Homogeneity	0.58	**0.98**	**1.00**	**0.94**	0.69	**1.00**	**0.98**	**0.97**
	Informational measure of correlation 1	0.53	**0.99**	**0.97**	**0.97**	0.65	**0.99**	**0.98**	**0.98**
	Informational measure of correlation 2	0.51	**0.98**	**0.95**	**0.96**	0.66	**0.95**	**0.97**	**0.94**
	Inverse difference moment normalized	0.61	**0.93**	**0.94**	**0.90**	0.68	**1.00**	**0.95**	**0.94**
	Inverse difference normalized	0.62	**0.96**	**0.98**	**0.93**	0.70	**1.00**	**0.96**	**0.96**
	Inverse variance	0.57	**0.98**	**1.00**	**0.94**	0.71	**1.00**	**0.98**	**0.97**
	Joint maximum	0.07	0.65	0.62	0.06	0.66	**0.96**	**0.93**	0.87
	Sum average	**0.98**	**0.95**	0.12	0.88	**0.99**	**0.99**	**0.99**	**0.99**
	Sum entropy	0.89	**0.98**	0.89	0.77	**0.99**	**0.98**	**0.99**	**0.98**
	Sum variance	**0.99**	**0.97**	0.26	0.90	**0.99**	**0.98**	**0.99**	**0.98**
	Joint variance	**0.92**	**0.93**	0.27	0.83	**0.98**	**0.99**	**0.99**	**0.99**
GLRLM (*n* = 11)	Gray level non-uniformity	0.69	0.69	**0.96**	0.70	**0.96**	**0.96**	**0.98**	**0.98**
	High gray level run emphasis	0.51	0.74	0.59	0.43	**0.99**	**0.98**	**0.98**	**0.98**
	Long run emphasis	0.51	**0.98**	**0.99**	**0.91**	0.54	**1.00**	**0.99**	**0.95**
	Long run high gray level emphasis	0.55	**0.99**	**0.99**	**0.92**	0.83	**0.99**	**0.98**	**0.98**
	Long run low gray level emphasis	0.54	0.52	0.24	0.50	0.72	**0.99**	**0.99**	0.89
	Low gray level run emphasis	0.58	0.53	0.14	0.52	0.73	**0.99**	**0.99**	**0.91**
	Run length non-uniformity	0.55	**0.98**	**0.99**	**0.91**	0.65	**0.99**	**0.99**	**0.96**
	Run percentage	0.54	**0.98**	**0.99**	**0.91**	0.62	**1.00**	**0.99**	**0.96**
	Short run emphasis	0.54	**0.98**	**0.99**	**0.91**	0.62	**1.00**	**0.99**	**0.96**
	Short run high gray level emphasis	0.65	**0.99**	**0.98**	**0.92**	**0.99**	**0.98**	**0.98**	**0.97**
	Short run low gray level emphasis	0.58	0.54	0.13	0.52	0.72	**1.00**	**0.99**	**0.90**

It is noted that two normalization methods were performed: mean ± 3SD and zero to maximum. Highly robust features (ICC > 0.9) are highlighted by bold text. *GLCM* Gray level cooccurrence matrix, *GLRLM* Gray level run length matrix, *ICC* Intraclass correlation coefficient, *SNR* Signal to noise ratio, *ROI* Region of interest, *N/A* Not applicable

**Table 3:** Number of features of high, moderate and low robustness in each feature class, as defined by average of intraclass correlation coefficient over 10 noise realizations, in reference to signal to noise variation with normalization of mean ± 3SD or zero to maximum

Feature group	High (ICC > 0.9)		Moderate (ICC 0.5–0.9)		Low (ICC < 0.5)	
	Mean ± 3SD	Zero to maximum	Mean ± 3SD	Zero to maximum	Mean ± 3SD	Zero to maximum
First order	11/13	11/13	2/13	2/13	0/13	0/13
GLCM	5/22	8/22	14/22	14/22	3/22	0/22
GLRLM	0/11	3/11	11/11	8/11	0/11	0/11

The denominator in the table signifies the total number of features in the feature class (i.e., first order, GLCM or GLRLM). *GLCM* Gray level cooccurrence matrix, *GLRLM* Gray level run length matrix, *ICC* Intraclass correlation coefficient

Second order texture features, namely GLCM and GLRLM, are impacted by the normalization procedure. The prior SNR analysis used mean ± 3 SD for normalization. Analysis was also performed by using zero to maximum normalization. Each method has its respective limitations. Mean ± 3SD normalization should be able to provide better separation due to a decrease in dynamic range, as compared to zero to maximum normalization making it more sensitive to small changes. However, mean ± 3SD is more likely to be sensitive to noise. Results using zero to maximum normalization procedure are summarized in Table 3 and Fig. 4b. First order features are not affected by normalization/quantization because they directly use all intensity value independently. As compared to the mean ± 3SD method, for GLCM features there is a trend toward higher ICC values, with no features in the low robustness group (ICC < 0.5). For GLRLM features, there is a similar trend, with higher proportion of features in the high robustness category (ICC > 0.9). As mentioned previously, Table 2 includes the full list of features and their respective ICC values. It is noted that in the ICC plots there is an observed clustering. It is hypothesized that these are because (1) a limited number of regions of interest are being compared, and (2) calculated features may be highly correlated.

### ROI delineation

In practice, intra- and inter-reader variability in the manual segmentation of regions of interest is inevitable. Subjective determination of abnormal tissue may not be consistent across readers due to variables such as difference in experience or difference in contrast windowing. The effect of ROI dilation and erosion was also studied to evaluate feature’s robustness to ROI variations.

**Fig. 5:**
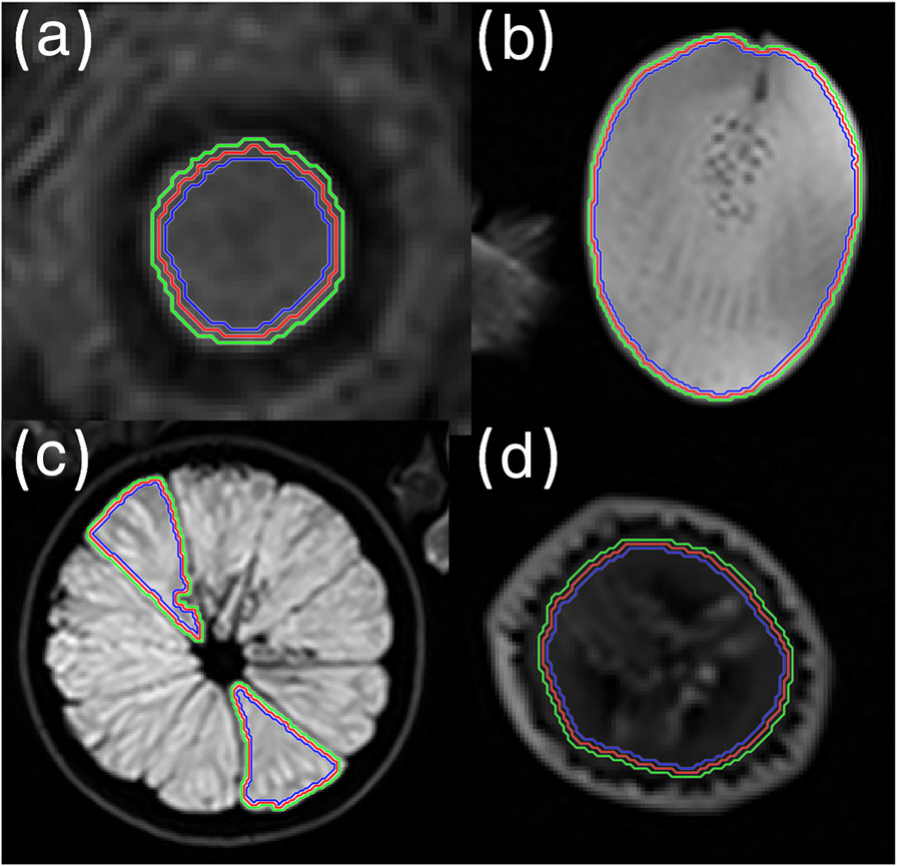
Dilation and erosion of region of interest (ROI), with the inner most (blue) ring being the eroded ROI, the center (red) ring being the original ROI and the outermost (green) ring being the dilated ROI for (**a**) pineapple core, (**b**) kiwi, (**c**) orange and (**d**) banana

Two types of ROI manipulations were performed: dilation (by 1 pixel) and erosion (also by 1 pixel) as shown in Fig. 5. Similar to above, analysis was performed using 2 different normalization techniques: mean ± 3SD and zero to maximum.

**Fig. 6:**
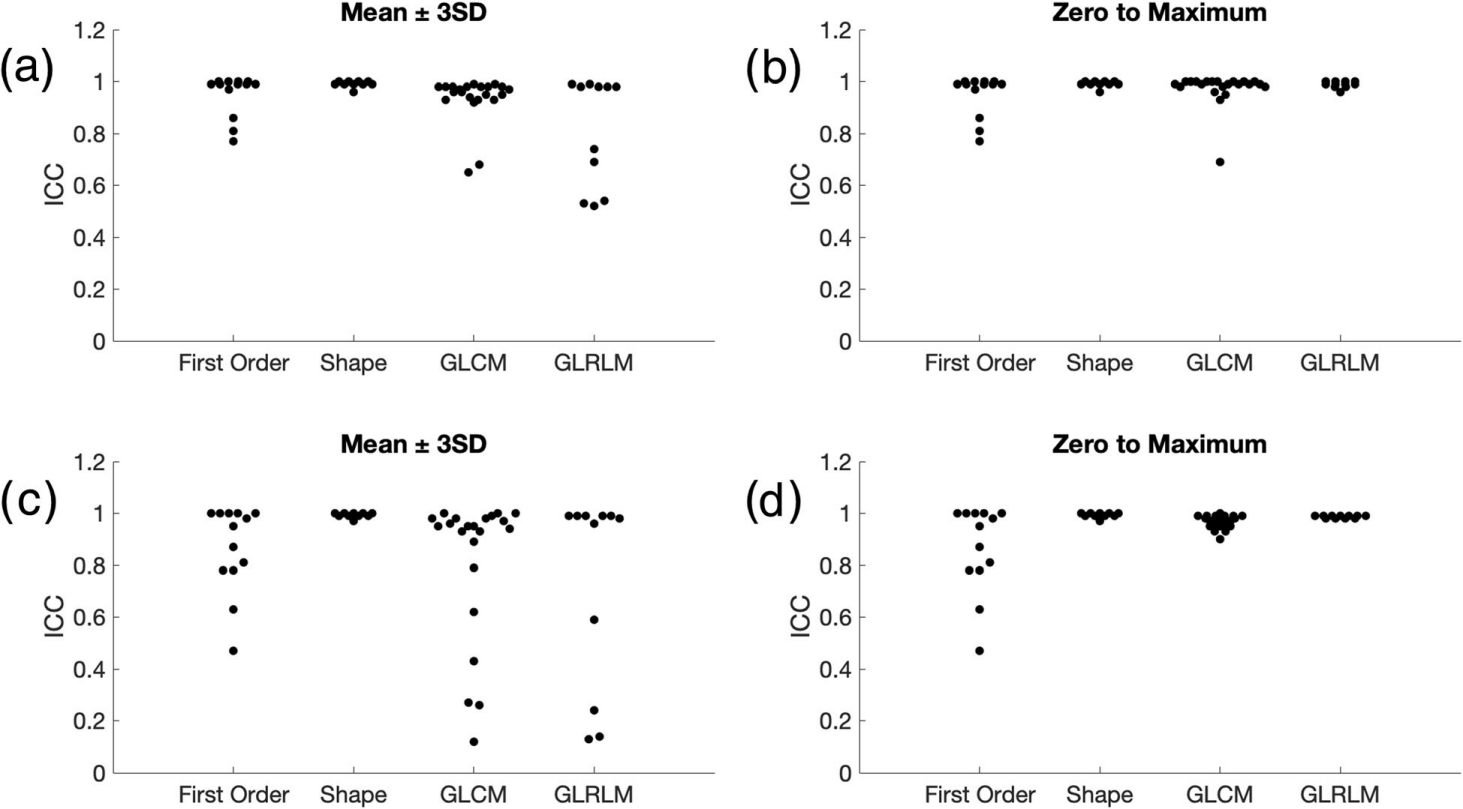
Average ICC over 10 noise realizations of first order, shape, GLCM and GLRLM features with (**a** and **b**) erosion of region of interest by one pixel with mean ± 3SD or zero to maximum normalization, respectively, and (**c** and **d**) dilation of region of interest by one pixel with mean ± 3SD or zero to maximum normalization, respectively.* ICC* Intraclass correlation coefficient, * GLCM* Gray level cooccurrence matrix, *GLRLM* Gray level run length matrix

For ROI erosion using mean ± 3SD normalization, results are summarized in Table 4 and Fig. 6a. All 10 shape features and 20 out of 22 GLCM features are found to be highly robust. However, only 10 out of 13 first order features and 6 out of 11 GLRLM features are found to be highly robust to ROI erosion. No feature is found to have an ICC less than 0.5. Results using zero to maximum normalization are summarized in Table 4 and Fig. 6b. By definition, first order and shape features are not affected by normalization differences. There is an upward trend in robustness of GLRLM feature, where all features are highly robust to ROI erosion using normalization method zero to maximum.

**Table 4:** Number of features of high, moderate and low robustness in each feature class, as defined by average of intraclass correlation coefficient over 10 noise realizations in reference to erosion of region of interest with normalization of mean ± 3SD or zero to maximum

Feature group	High (ICC > 0.9)		Moderate (ICC 0.5–0.9)		Low (ICC < 0.5)	
	Mean ± 3SD	Zero to maximum	Mean ± 3SD	Zero to maximum	Mean ± 3SD	Zero to maximum
First order	10/13	10/13	3/13	3/13	0/13	0/13
Shape	10/10	10/10	0/10	0/10	0/10	0/10
GLCM	20/22	21/22	2/22	1/22	0/22	0/22
GLRLM	6/11	11/11	5/11	0/11	0/11	0/11

The denominator in the table signifies the total number of features in the feature class (i.e., first order, shape, GLCM or GLRLM). *GLCM* Gray level cooccurrence matrix, *GLRLM* Gray level run length matrix, *ICC* Intraclass correlation coefficient

For ROI dilation, mean ± 3SD normalization results are summarized in Table 5 and Fig. 6c. Shape is a highly robust feature. However, the other feature categories have relatively poorer robustness, with only 7 out of 13, 15 out of 22 and 7 out of 11 features with ICC greater than 0.9 for first order, GLCM and GLRLM groups, respectively. Table 2 lists individual features and their respective ICC values. Zero to maximum normalization results are summarized in Table 5 and Fig. 6d. There is an upward trend of ICC values using zero to maximum normalization method. Similar clustering is observed within ICC plots as described previously.

**Table 5:** Number of features of high, moderate and low robustness in each feature class, as defined by average of intraclass correlation coefficient over 10 noise realizations in reference to dilation of region of interest with normalization of mean ± 3SD or zero to maximum

Feature group	High (ICC > 0.9)		Moderate (ICC 0.5–0.9)		Low (ICC < 0.5)	
	Mean ± 3SD	Zero to maximum	Mean ± 3SD	Zero to maximum	Mean ± 3SD	Zero to maximum
First order	7/13	7/13	5/13	5/13	1/13	1/13
Shape	10/10	10/10	0/10	0/10	0/10	0/10
GLCM	15/22	21/22	3/22	1/22	4/22	0/22
GLRLM	7/11	11/11	1/11	0/11	3/11	0/11

The denominator in the table signifies the total number of features in the feature class (i.e., first order, shape, GLCM or GLRLM). *GLCM* Gray level cooccurrence matrix, *GLRLM* Gray level run length matrix, *ICC* Intraclass correlation coefficient

As expected, dilation resulted in poorer robustness when compared to erosion. This is because dilation may incorporate tissue that is outside the ROI, whereas erosion still only includes voxels in the original ROI. It is noted that in our study dilation of the ROI may include "fruit skin", which can be highly different in visual appearance than the interior, or surrounding air. In non-phantom study, such as a ROI of a tumor, the overestimation or dilation of an ROI would likely include surrounding tissue and not surrounding air. However, there are tumors which are located next to air cavities, such as nasopharyngeal cancer, and robustness of features to dilation may be application based. The result of this comparison indicates that it may be more beneficial to be conservative when defining an ROI.

### Small voxel size variation

In order to accommodate the different sizes of patients, it is a general practice for the technologist to adjust the field of view (FOV) on the fly without changing other parameters. Although strictly speaking, changing FOV will always affect some other parameters such as TE, bandwidth, gradient slew rate, which in turns affecting SNR. The effect of these small voxel size variations, and its relation to radiomic feature robustness, is understudied. In this part of the study, variation of voxel size was introduced by acquiring images with slight change of the FOV and matrix size. To remove effect of SNR variations caused by pixel size changes, all images were normalized to the same SNR. Previous studies have tried to solve this problem by performing interpolation, however, interpolation introduces other complications and affect feature robustness [27].

**Fig. 7:**
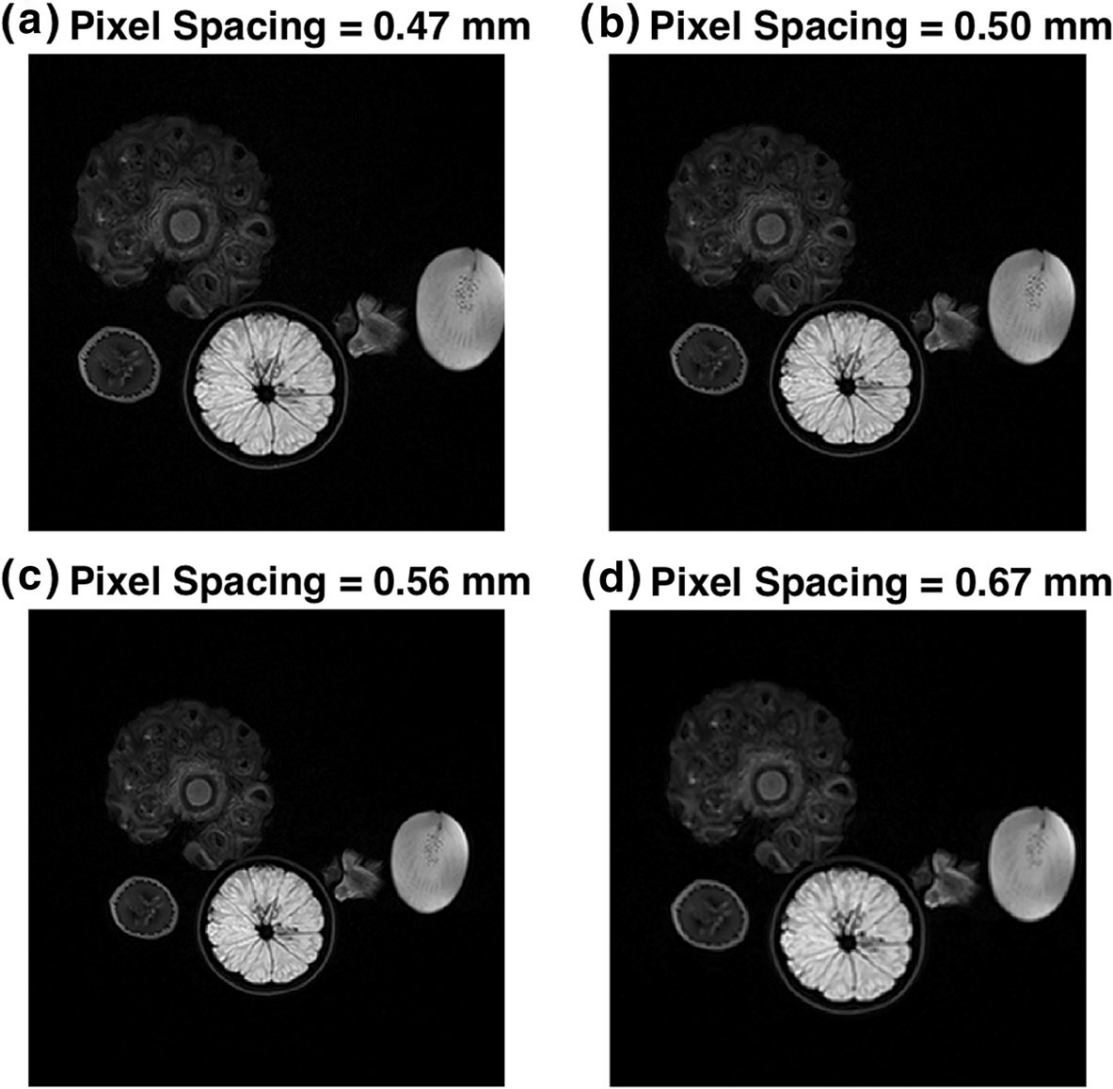
Image of small variation in pixel size achieved by changes in acquisition parameters: (**a**) 0.47 mm, (**b**) 0.50 mm, (**c**) 0.56 mm and (**d**) 0.67 mm

**Fig. 8:**
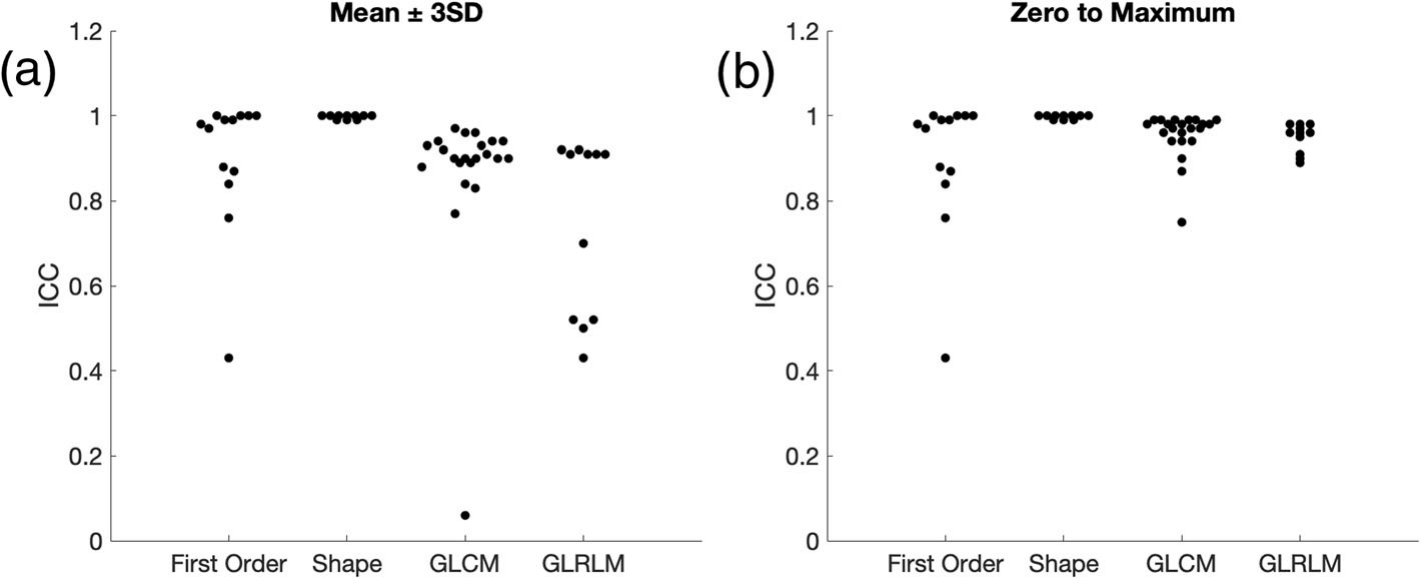
Average ICC over 10 noise realizations of first order, shape, GLCM and GLRLM features with small variation in voxel size with (**a**) mean ± 3SD and (**b**) zero to maximum normalization for voxel size variation. *ICC* Intraclass correlation coefficient, *GLCM* Gray level cooccurrence matrix, *GLRLM* Gray level run length matrix

The same slice was acquired with 4 different in-plane resolutions of 0.47, 0.50, 0.56 and 0.67 mm as shown in Fig. 7a-d, respectively. All other parameters were kept the same when possible. The SNRs of individual images were normalized to an SNR level of 75 by adding Gaussian noise and 10 different noise realizations were performed numerically. Results with mean ± 3SD normalization are summarized in Table 6 and Fig. 8a. Even though minor voxel size variation will affect ROI, which in turn affects shape features, all shape features were found to be robust to minor voxel size variations. First order, GLCLM and GLRLM features groups are found to have 8 out of 13, 12 out of 22 and 6 out of 11 features, respectively, to be highly robust to small differences in voxel sizes. Individual feature ICCs are reported in Table 2. Results for zero to maximum normalization are summarized in Table 6 and Fig. 8b. Similar upward trends in ICC of GLCM and GLRLM are noted. Similar clustering is observed within ICC plots as described previously.

**Table 6:** Number of features of high, moderate and low robustness in each feature class, as defined by average of intraclass correlation coefficient over 10 noise realizations in reference to pixel size with normalization of mean ± 3SD or zero to maximum

Feature group	High (ICC > 0.9)		Moderate (ICC 0.5–0.9)		Low (ICC < 0.5)	
	Mean ± 3SD	Zero to maximum	Mean ± 3SD	Zero to maximum	Mean ± 3SD	Zero to maximum
First order	8/13	8/13	4/13	4/13	1/13	1/13
Shape	10/10	10/10	0/10	0/10	0/10	0/10
GLCM	12/22	19/22	9/22	3/22	1/22	0/22
GLRLM	6/11	10/11	3/11	1/11	2/11	0/11

The denominator in the table signifies the total number of features in the feature class (i.e., first order, shape, GLCM or GLRLM). *GLCM* Gray level cooccurrence matrix, *GLRLM* Gray level run length matrix, *ICC* Intraclass correlation coefficient

Small variability in voxel size does not result in a large visual difference, however differences are observed in radiomic feature extraction as reported here. Since small variation in voxel size can result in a reduction in robustness, it is expected that this result is even more concerning when comparing voxel sizes of larger differences. Especially in multi-institutional studies, it is common to see a large range of different voxel sizes used in analysis.

### Limitations

Our study has several limitations. Firstly, the results from phantom study cannot always be transferred to clinical studies. However, we note that robustness of radiomic features are application dependent and phantoms can still be used to investigate feature pre-selection pipeline. One way to show the transferability of phantom study is to compare the variability of each feature obtained from phantom to that calculated from tumors [60]. Secondly, we investigated only one sequence from one particular scanner. Although there are fundamental differences between scanners, inter-scanner variability could be addressed if the bias is corrected in image preprocessing step [51]. Lastly, we only investigated 2D radiomic features of certain classes. Future work should explore robustness of 3D features including filter-based features from multi-scanner images combined with clinical data.

## Conclusions

Radiomic analysis is a step towards personalized medicine by an exponential increase in the amount of quantitative data that can be extracted from medical images. In current literature, feature robustness in MRI is understudied and feature extraction techniques are not universally standardized. There is a need for systematic evaluation of feature robustness. This is required to ensure that a predictive biomarker is reproducible and generalizable, especially across different institutions where parameters can be very variable. Application-based feature pre-selection step will be pivotal in anticipation for incorporation of radiomics-based tools in the clinic.

## Methods

### Phantom MR imaging

A pineapple, a gold kiwi, an orange, a banana and a strawberry placed on Styrofoam box served as radiomics phantom for our study. All images were acquired on a 3 T Siemens scanner (Biograph mMR) with a T2-weighted Turbo Spin Echo sequence using a 12 channel PET compatible head-coil. Acquisition parameters: echo train length = 18, TE = 98 ms, TR = 7360 ms, slide thickness/gapping = 2/0 mm, pixel bandwidth = 219 Hz, flip angle = 150 degree, 100% phase sampling, 100% phase FOV, body coil transmission, 1 average. Different axial resolutions were acquired by changing matrix size and FOV with parameters listed in Table 7.

**Table 7:** Voxel size, matrix size and field of view used in the voxel size variation analysis

Series	Voxel size (mm)	Matrix size	FOV (mm)
1	[0.47,0.47,2]	512 × 512	240
2	[0.50,0.50,2]	512 × 512	256
3	[0.56,0.56,2]	512 × 512	288
4	[0.67,0.67,2]	384 × 384	256

*FOV* Field of view

#### Image segmentation

First, image segmentation was performed manually on one slice of Series 2 using ITK-SNAP (version 3.6.0; http://www.itksnap.org). The ROIs on different fruits were then interpolated with linear method on the same slice of the rest of the series using MATLAB R2019a. To be conservative with ROI, threshold was set to 1. All interpolated ROIs were visually checked and corrected manually to exclude the fruit/air interface and discontinuities.

### Image processing

In order to calculate the SNR of the original image the mean intensity of a homogenous region within a ROI (kiwi) is divided by the mean intensity of the background. These ROIs are shown in Fig. 2b. Because the mean of a Rayleigh distribution is $\sqrt{\pi /2}\ \sigma$, where *σ* is the mode, the calculated SNR was further corrected by dividing $\sqrt{\pi /2}$ . Complex Gaussian noise was added to the original image and magnitude images were used for the analysis. Two noise levels (SNR 45 and 75) were generated from the original image whose SNR is 124. Ten different noise realizations were performed numerically for each SNR level in order to identify the results with test-retest imaging. In-built MATLAB *imdilate* and *imerode* functions with a 3*3 stucturing element were used to dilate and erode ROIs. The entire preprocessing was implemented in MATLAB (MATLAB R2019a).

### Feature extraction

A set of 56 features were extracted using an IBSI compliant in-house software (in MATLAB) partially adapted from the Vallieres radiomics toolbox [61] and ImFEATbox [62]. Features are summarized in Table 2. Thirteen of the features were first order statistics based, 10 were 2D shape based, while texture features were computed from the grey-level co-occurrence matrix (GLCM, 22 features) and grey-level run-length matrix (GLRLM, 11 features) merged from all four 2D directional matrices. The definitions of first order statistics based and texture features could be found in Parmar et al [63], while the definitions of 2D shape features could be found in Griethuysen et al [64]. Both first order and 2D shape features were directly implemented in MATLAB based on their definitions. For texture features, GLCM and GLRLM matrix computation and GLRLM feature extraction was adapted from the Vallieres radiomics toolbox, while GLCM features were adapted from ImFEATbox based on their definitions. Prior to calculating texture matrix, all images underwent intensity discretization to 64 levels based on IBSI recommendations, with intensity values rescaled by mean ± 3SD or zero to maximum intensity (to assess texture feature robustness on different discretization scales).

### Robustness analysis

Feature robustness was assessed using ICC when performed at different SNR, different acquisition voxel size and ROI transformation, assuming these variations possess no consistent bias for different ROIs. Each noise level, voxel dimension and ROI transformation accounts for a rater and each intensity mask (containing intensities with selected voxels) accounts for a subject. Based on ICC reporting guidelines [65], ICC (2,1) was selected (“2-way mixed-effects model, single rater, absolute agreement”) as features are considered to be stable if their values remain the same across different variations. ICCs were calculated in MATLAB (MATLAB R2019a). For SNR and ROI dilation/erosion analysis, 5 ROIs were analyzed for a single image resolution (0.5 mm × 0.5 mm × 2.0 mm), with 10 different noise realizations, resulting in 50 samples per image. There were 2 groups being compared (SNR = 45 versus SNR = 75, original ROI versus eroded ROI, original ROI versus dilated ROI). For voxel size analysis, 5 ROIs were analyzed with 10 different nose realizations, resulting in 50 samples per image. These were analyzed across 4 different in-plane resolutions (0.47, 0.50, 0.56, 0.67 mm). ICC was assessed between groups for each calculated feature.

## Data Availability

All data will be provided upon written request.

## Abbreviations

1-NN: 1-nearest neighbor
2D: Two-dimensional
ACC: Average correlation coefficient
ADC: Apparent diffusion coefficient
CCC: Concordance correlation coefficient
CT: Computed tomography
CV: Coefficient of variation
DCE: Dynamic contrast-enhanced
DISCO: Differential subsampling with cartesian ordering
DR: Dynamic range
DSC: Dice similarity coefficients
DWI: Diffusion-weighted imaging
FLAIR: Fluid-attenuated inversion recovery
FOV: Field of view
GLCM: Gray level cooccurrence matrix
GLRLM: Gray level run length matrix
IBSI: Image biomarker standardization initiative
ICC: Intraclass correlation coefficient
k-NN: k nearest neighbor
LDA: Linear discriminant analysis
LoG: Laplacian of Gaussian
MRI: Magnetic resonance imaging
N/A: Not applicable
NAs: Number of acquisitions
PDW: Proton density weighted
PE: Peak enhancement
PET: Positron emission tomography
POE: Probability of error
ROC: Receiver operating characteristic
ROI: Region of interest
SBW: Sampling bandwidth
SER: Signal enhancement ratio
SNR: Signal to noise ratio
TE: Echo time
TR: Repetition time
wCV: Within-subject coefficient of variation
